# Biobased Compostable Plastics End-of-Life: Environmental Assessment Including Carbon Footprint and Microplastic Impacts

**DOI:** 10.3390/polym16213073

**Published:** 2024-10-31

**Authors:** Anthony Keyes, Christopher M. Saffron, Shilpa Manjure, Ramani Narayan

**Affiliations:** 1 Northern Technologies International Corporation, Circle Pines, MN 55014, USA; 2 Department of Chemical Engineering and Materials Science, Michigan State University, East Lansing, MI 48824, USA; saffronc@msu.edu

**Keywords:** carbon footprint, life-cycle assessment, bio-based, compostable, microplastics, carbon sequestration

## Abstract

In this paper, we examine how traditional life-cycle assessment (LCA) for bio-based and compostable plastics overlooks issues surrounding carbon sequestration and microplastic persistence. To outline biased comparisons drawn from these omitted environmental impacts, we provide, as an example, a comparative LCA for compostable biobased vs. non-compostable fossil-based materials. In doing so we (1) demonstrate the proper way to capture carbon footprints to make fair comparisons and (2) identify the overlooked issues of microplastics and the need for non-persistent alternatives. By ensuring accurate biogenic carbon capture, key contributors to CO_2_ evolution are properly identified, allowing well-informed changes to formulations that can reduce the environmental impact of greenhouse gas emissions. In a complimentary manner, we summarize the growing research surrounding microplastic persistence and toxicity. We highlight the fundamental ability and the growing number of studies that show that industrial composting can completely mineralize certified compostable materials. This mineralization exists as a viable solution to combat microplastic persistence, currently an absent impact category in LCA. In summary, we propose a new paradigm in which the value proposition of biobased materials can be accurately captured while highlighting compostables as a solution for the increasing microplastic accumulation in the environment.

## 1. Introduction

In recent years, bio-based and compostable plastics have seen a rise in both global consciousness and industrial production. In 2021, the growth of bio-based and biodegradable plastics was estimated to increase by a factor of three times from 2.4 to 7.5 million tons produced annually [1]. Additionally, the land used to grow the renewable feedstock to produce bioplastics in 2021 only accounts for just over 0.01 percent of the global agricultural area (5 billion hectares). As a major goal for bio-based plastics is founded on replacing their fossil-based counterparts, discussions surrounding these materials perpetuate an underlying need to make comparisons. While the standards to compare these plastics in both mechanical and chemical performance are largely equivalent, the discussion around their environmental impacts has seen much more discourse.

A recent review evaluating LCAs of pure fossil-based and bio-based plastics found that the variation in impacts found between different studies for identical polymers was as high as 400% [2]. The conclusions drawn from the review were to adopt the guidelines of the EU Product Environmental Footprint (PEF) to reduce this variation, allowing for more fair comparisons. While a consistent set of impact measurements would lead to less variation between identical fossil-based or bio-based plastics, the comparison that takes place between a fossil-based plastic versus bio-based plastic would not necessarily be fairer in evaluation. This observation has been brought up by the European Bioeconomy Alliance (BPA), which has the ways in which the Joint Research Center’s (JRC’s) LCA does not allow for fair comparisons between fossil-based and bio-based plastics listed [3]. Their concern was due to the way in which biogenic carbon was captured in the system boundaries. Even with wider adoption by the European Commission to set the EU PEF as guidance for environmental impact assessment, the comparisons between fossil-based and bio-based plastics have still been recognized as unfair toward bio-based materials [4]. The contentious issue is the lack of benefit granted for biogenic carbon at the beginning of the life cycle. This benefit is granted in other LCA methodologies such as the ISO 22526 standards, which account for the carbon sequestration of biobased materials at the beginning of the life cycle [5].

This contrast in calculating the environmental impact is not ideal when deciding on whether a bio-based product is worth replacing a fossil-based one, as they can lead to vastly different outcomes for an identical material. A real-world example was selected for this work to capture this difference. Following the calculation of environmental impacts, we highlight why accurate capturing of biogenic carbon should be the standard to allow for sound comparison of bio-based and fossil-based plastics. Moreover, we expand the discussion to end-of-life realities as they relate to microplastics.

Within the past few years, research has come out showing the accumulation of microplastics in human blood [6], the lungs [7], the brain [8], and even crossing the placental membrane [9]. While the discussion around plastic waste has largely focused on bulk material, the fraction of microplastics that contribute to the total weight of plastic accumulation in the world is estimated to reach 13.2% by 2060 [10]. We anticipate that the recent advances in the detection and characterization of microplastics will unquestionably lead to more discussions about how to prevent the accumulation of these materials in the environment. As such, the benefits of composting as a value proposition should be captured in an LCA, as industrial composting standards (EN 13432) [11], when followed, have been shown not to contribute to microplastic generation [12]. While current LCA methodologies do not capture the impact of microplastics, the recent detection in humans and quantification of toxicity serves as the impetus for impact categories that account for this reality of plastic accumulation in the environment. This work serves to first clarify the discourse surrounding fair comparisons of bio-based and fossil-based plastics, while secondly capturing the difference in compostable vs. non-compostable products as they relate to persistence and mineralization. We envision this real-world example to serve as a model to fairly compare fossil-based and bio-based plastics with one another, while also engendering broader discussions surrounding the relationship between composting and microplastics.

## 2. Materials and Methods

**Figure 1:**
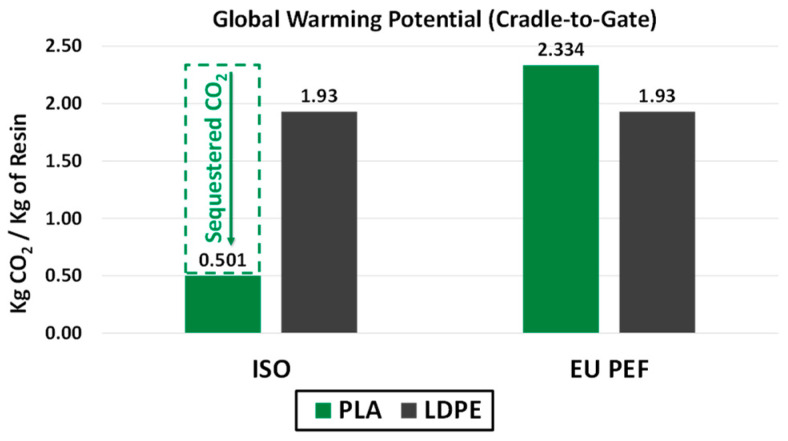
Global warming potential for PLA and LDPE resins utilizing the ISO standard and the EU PEF guidance. Although both treat fossil-based resins the same, bio-based resins vary largely due to whether biogenic carbon is captured (ISO) or omitted (EU PEF).

An inherent understanding of the value proposition of bio-based and compostable plastics is depicted in Figure 1, highlighting either their renewability or end-of-life realities. As bio-based materials are created on a much shorter timescale than fossil fuel reserves, bio-based polymers are characterized as a form of carbon sequestration. This means that bio-based polymers can be said to have a lower feedstock carbon emission burden than fossil-based alternatives. As for compostable materials, their ability to break down into biomass and mineralize into CO_2_ and H_2_O in the presence of microorganisms provides a solution to many plastic waste issues. For example, landfilling is a common problem when plastics are contaminated with food, as recycling of the materials is not possible.

While these benefits catalyze and propel initial discussions for the switch from fossil-based and/or non-compostable plastics, the environmental impacts must be accurately presented in these discussions. A comparison of environmental impacts is largely dependent on LCAs, which currently serve as the prevailing method to evaluate this class of materials. Fair comparisons must be at the core of LCAs for fossil-based vs. bio-based and non-compostable vs. compostable plastics, as the inherent benefits will only be adopted should the environmental impacts be fairly illustrated. For this article, a focused discussion of biogenic carbon as it relates to bio-based materials is established and reveals how end-of-life scenarios can dictate its use when compared to fossil-based materials. Herein, an LCA comparing polyethylene gloves (fossil-based, non-compostable) with those made from commercial Natur-Tec^®^ BF700Z-X resin (bio-based, compostable) utilizing both ISO and EU PEF methodologies is performed. Attention is specifically given to how global warming potential varies greatly for the ISO 22526:2020 standards and the current EU PEF guidance. Lastly, consideration for plastic persistence and microplastic accumulation is shown, highlighting impacts currently not captured in either LCA methodology. The goal of this real-world example is to provide a template for fair comparisons regarding fossil-based and bio-based materials, while also expanding the discussion of compostable materials from a mere remedy for landfilling and outlining the overlooked benefits respective to the rise in microplastic pollution.

## 3. Results

The LCA was conducted based on the ISO 14040:2006, ISO 14044:2006, and ISO 22526:2020 standards [5,13,14]. Additionally, the EU PEF guidance for global warming potential is demonstrated on identical systems to reveal the clear lack of biogenic carbon capture. When biogenic carbon is not accurately accounted for, the value proposition of bio-based resins is lost and can lead to a bias toward fossil-based materials as shown in Figure 1. The sequestration of CO_2_ for PLA reduces its GWP by ~79% [15]. As such, the LCA in this report was conducted utilizing ISO standards so that a fair comparison could be achieved between fossil-based and bio-based materials, which is further discussed in Section 3.4.

### 3.1. Goal and Functional Unit

The goal of this case study was to perform a comparative LCA to quantify the advantages and disadvantages of a bio-based and compostable alternative to traditional fossil-based and non-compostable gloves used in the food industry. The function of the gloves is to serve as protection and a barrier, especially for food handling purposes. Disposable plastic gloves are major contributors to plastic waste production, and those that are contaminated with food are not suitable for recycling as an end-of-life opportunity [16,17]. As such, composting was selected as an end-of-life scenario as it is not sensitive to food contamination, which also addresses the plastic waste issues linked to landfilling. While the switch to compostable gloves was to address the plastic waste issue, it was important to examine other environmental impacts that were affected by this shift. As such, eight impact categories, which are recognized by both ISO and the EU PEF, were selected and measured for both fossil-based and bio-based gloves.

The two systems were compared using a functional unit of 1,000,000 gloves, i.e., both systems produce 1,000,000 gloves and thus provide equivalent service in the end market. As the materials are not chemically equivalent, the mass associated with the functional unit is not identical (See Supporting Information). The filmmaking process is set in India and the resin supply was determined based on supply chain partners in the USA, Asia, and Europe. The time horizon for this study is set to 365 days, which would be long enough to account for industrial composting (180 days) as an end-of-life scenario for the compostable gloves.

### 3.2. Product Systems

LDPE resin is used to make gloves (hereto referred to as LDPE glove); the main resin input is fossil-based polyethylene. To capture the environmental impacts of producing the resin, a cradle-to-gate assessment based on the analysis of LDPE was utilized [18] in addition to an analysis of HDPE [19]. For BF700Z-X resin used to make gloves (hereto referred to as NT gloves), the main resin inputs consist of PLA (biobased, compostable) and a copolyester (fossil-based, compostable). The data for PLA were obtained from a literature assessment of Luminy PLA produced from sugarcane in Thailand [15]. As such, the cultivation, harvesting, and production of PLA are all captured in one stream input. For the copolyester resin utilized for the gloves, a confidential report cataloging the impact categories was obtained from the supplier of the resin.

For transportation, calculations were based on the distance between ports and facilities. The life cycle inventory table shows the calculated distances measured, as well as the assumption of what type of transportation would be utilized. Short haul trucks were employed for distances less than 200 km, long haul trucks for distances greater than 200 km, freight trains when available, as well as shipping when traveling from country to country. For truck and freight calculations, the greenhouse gases, and regulated emissions, the energy use in technologies (GREET^®^) model was employed for well to wheel (W2W) calculations [20]. As for ship emissions, data were collected from an LCA book chapter on ship emissions [21].

### 3.3. Scope

**Figure 2:**
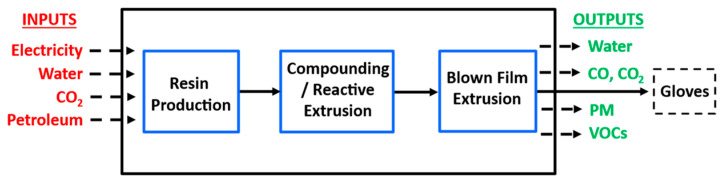
General overview of the boundaries explored for glove making. The resin inputs, extrusion process, and transportation are all tracked, with their emissions recorded and compared.

Figure 2 shows a simplified system boundary that depicts how the glove making processes were investigated. All inputs and outputs are detailed in Supporting Information Tables S1–S4. As mentioned above, this LCA covers eight impact categories including global warming, marine eutrophication, terrestrial eutrophication, acidification, particulate matter, renewable and non-renewable energy use, and ozone depletion potential. As gloves are the sole product of this LCA and gloves are the only end products intended for manufacture, no co-product allocation of burdens is necessary. Therefore, all environmental benefits and burdens are assigned to the production of 1,000,000 gloves.

### 3.4. Impact Categories and Assessment Methods

The LCIA phase of the LCA quantifies the environmental impacts of the various emissions that were compiled in the life cycle inventory phase. All assumptions made are transparently documented and justified within this report. Recognizing the inevitable variation in data quality, data quality indicators were assigned to each data element using the five-factor Weidema method, with scores shown in Tables S5 and S6. This information was obtained from the EPA’s Guidance on Data Quality Assessment for Life Cycle Inventory Data [22].

In the absence of credible and available data, assumptions were made to allow for a faithful portrayal of the systems. The main assumptions are listed below:

1. Additives such as slip and color agents did not have data related to their impacts. Since the additives are used in both NT gloves and LDPE and are less than 2% by weight, they were drawn outside the system boundaries. For the NT glove case, modifying agents were also removed from the system boundaries as the material contributes less than 0.5% by weight to the formulation, and the functional groups are not known to have a noticeable impact on the measured impact categories;
2. After the blown film extrusion process, the transportation and energy demand costs are assumed to be the same for the NT gloves and LDPE gloves, and as such, have been drawn outside the system boundaries;
3. Environmental emissions for resin manufacturing processes were taken from LCAs and supplier models, evaluating them in a cradle-to-gate approach. The impact categories measured for LDPE, copolyester, and PLA, were assumed to scale linearly and were reduced to unit inputs in stream tables (Supporting Information Tables S1–S4);
4. LDPE supply was assumed to come from within India as that is where the formulation would be made, and Reliance Industries was selected as a model supplier to obtain the transportation distance. Additionally, the synthesis of LDPE from reliance was equated to the report conducted in America for LDPE production [18];
5. HDPE data were used to calculate particulate matter and terrestrial eutrophication values for the LDPE gloves [19]. The measured global warming potential for both reports was similar, 1.89 and 1.90 kg CO_2_/kg of material, respectively, and the other impact categories were also expected to be similar in terms of emissions;
6. US data for emissions per citizen were used to normalize the impact categories per emissions compared to a US citizen over the course of a year to determine the significance of associated emissions (Figures S3 and S4);
7. Transport of the film to make the gloves and for distribution of the gloves is the same for both processes and left outside the system boundaries.

### 3.5. Systen Boundaries

**Figure 3:**
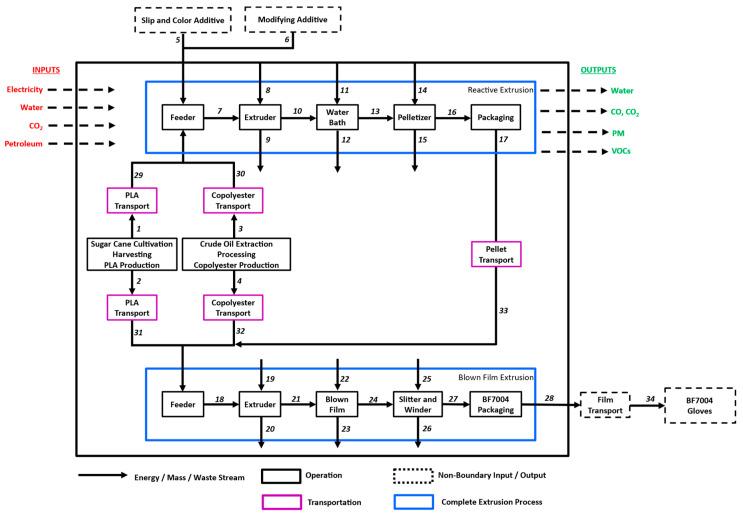
System boundaries for NT glove making. Numbers correspond to stream Tables S1 and S2 in the Supporting Information.

**Figure 4:**
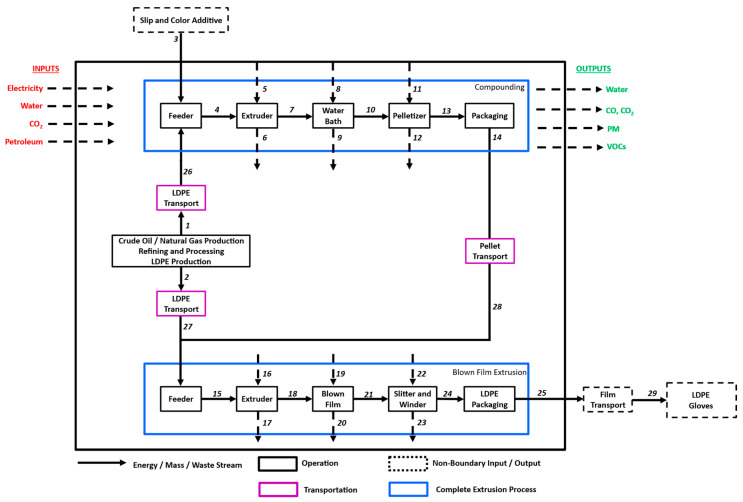
System boundaries for LDPE glove making. Numbers correspond to stream Tables S3 and S4 in the Supporting Information.

Detailed system boundaries for the NT gloves and LDPE gloves are shown in Figure 3 and Figure 4. The stream tables for each operation are accounted for (Supporting Information Tables S1–S4). This cradle-to-gate system boundary was used starting with the production of the resin and ending at the final film making. The glove making process is excluded from this analysis, which would be the same for all product systems. Additionally, the consumer use phase was not captured, and a larger discussion was had around the end-of-life realities in Part Two of this article.

### 3.6. Impact Assessment

**Figure 5:**
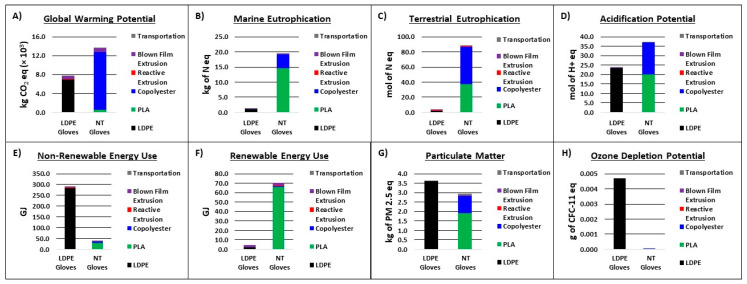
Comparative analysis of LDPE and NT gloves in the eight impact categories covered for this LCA.

The life cycle impact assessment (LCIA) phase of the LCA quantifies the environmental impacts of the various emissions that were compiled in the life cycle inventory phase. In this study, the LCIA was completed for eight impact categories: global warming potential, marine eutrophication, terrestrial eutrophication, acidification potential, particulate matter, non-renewable energy use, renewable energy use, and ozone depletion potential. The impact categories are fully tabulated in the appendix section for both NT gloves and LDPE gloves. Figure 5 shows the stacked bar charts that break down emissions into component contributors such as transportation, blown film extrusion, reactive extrusion (compounding for LDPE), and resin emissions. The impact categories for all the resins were used directly from other LCA data, and as such, no calculation from individual streams was performed. For transportation and extrusion processes, the Tool for Reduction and Assessment of Chemicals and Other Environmental Impacts (TRACI) model was employed to convert single emissions into equivalent impact measurements [23].

A detailed summary for each individual impact category measured in Figure 5 is detailed in the Supporting Information. Sensitivity analyses were also performed to show consistency in the collected data. In typical comparative LCA assessments, the data collection would end here, and decisions based on the benefits of each product would be carried out. If conclusions were drawn at this point, NT gloves would be seen as a larger contributor to GWP based on material inputs, while microplastic issues known to arise from plastic products would go unnoticed. To contest this narrow comparative outlook, the next two sections seek to reveal the too often overlooked additive value of biobased/compostable products as it relates to biogenic carbon and microplastic persistence.

## 4. Discussion

### 4.1. Accounting for Biogenic Carbon

**Figure 6:**
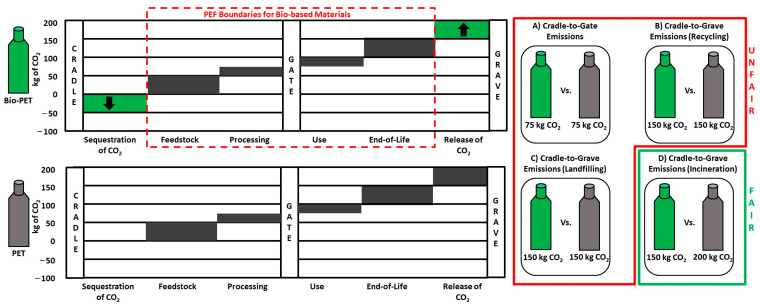
A representation of the lack of fair comparisons for carbon emissions when comparing bio-based and fossil-based materials utilizing current EU PEF methodologies. The emissions were made identical for both materials to make it apparent how fossil-based materials are favored with this type of comparison. (**A**–**C**) Comparisons made that do not highlight the benefits of bio-based materials as the sequestered carbon is not captured. (**D**) The only example where the benefits of biobased materials are displayed is when the fossil-based material is incinerated.

While accounting for the energy and mass flow within the system boundaries (Figure 3 and Figure 4), attention was afforded to account for the carbon footprint associated with the resin material themselves based on ISO 22526:2021 standards (Table 1). For this calculation, the weight percentage of carbon was calculated for each resin blend, 60% vs. 86% for NT gloves and LDPE, respectively. Once the percent carbon content was calculated, the percent of biobased content was then calculated to account for the sequestration of CO_2_. The NT gloves contain 21% of bio-sourced carbon, while LDPE has no biogenic content. The higher carbon content in combination with the lack of sequestration made LDPE 49% higher than NT gloves in terms of CO_2_ evolution when comparing resins. These data, in combination with the GWP (Figure 1), highlight how proper sequestration of biogenic carbon is necessary to capture the value of bio-based plastics compared to fossil-based plastics. The EU PEF states that “removals and emissions of biogenic carbon sources shall be kept separated in the resource use and emissions profile” [24]. The reason this approach skews heavily in favor of fossil-based materials is because the sequestration is forcibly tied to the assumed emissions for a bio-based material. In Figure 6, this method of accounting for CO_2_ emissions falls apart depending on the system boundaries in the example of PET and bio-PET. For this example, we assume that the emissions associated with the production of bio-PET and PET were identical, with the only difference being that one is bio-based and the other fossil-based. Even though the bio-PET sequesters 50 kg of CO_2_ per functional unit, there are limited amounts of comparison where the bio-based PET would have benefits. Taking Figure 6A, for example, where the cradle-to-gate for both bottles is shown, without considering the 50 kg of CO_2_ sequestered by bio-PET, both bottles would have identical emissions of 75 kg of CO_2_. From a cradle-to-gate perspective, the benefits of a bio-based material are completely hidden. Even when extending to a cradle-to-grave approach, the benefits of bio-based materials are still not represented in cases of recycling (Figure 6B) and even landfilling (Figure 6C). Only when the fossil-based material is incinerated (Figure 6D) and releases CO_2_ would the benefits be noticeable for a comparison of the bio-based and fossil-based material.

From a commercial perspective, this problem is exacerbated when LCA documents are required for a material whose end-of-life is varied and unknown, as is the case for plastic resins. When the end-of-life is not singular for manufactured goods, commercial products are evaluated from a cradle-to-gate perspective. As we have shown above, the EU PEF method would not account for the benefit of a bio-based material when compared to a cradle-to-gate method. Even when LCAs are conducted from a cradle-to-grave perspective, if the commercial product is long-lasting (such as a plastic bottle, which is recycled or landfilled), the benefits are still not captured. Considering this, it is imperative that the EU PEF methodology is updated to equal those of ISO 22526, ISO 14067, EN 15804, EN 16760, or other LCA standards that clearly document biogenic carbon, allowing for fair comparisons regardless of system boundaries and end-of-life scenarios [5,25,26,27]. From herein, we compare LDPE and NT gloves utilizing ISO standards, which grants this fair comparison in a cradle-to-gate perspective.

### 4.2. Microplastics: Sources and Environmental Accumulation

LCA practitioners are only starting to include midpoint and endpoint impacts for microplastics. A literature review in September of 2024 using ISI Web of Science only returns seven articles when using the title search terms “microplastics and (LCA or lifecycle or life cycle)”. In work by Corella-Puertas et al. [28,29], characterization factors for polystyrene and tire particles were developed to assess the “physical effects on biota” impact category. In this category, the comparative toxic unit for aquatic ecotoxicity was related to the potentially affected fraction of species to construct a midpoint characterization factor. To construct an endpoint characterization factor, the midpoint characterization factor is multiplied by a severity factor, which accounts for the potentially disappeared fraction of species, followed by dividing this quantity by water depth. Microplastic degradation and sedimentation are especially important mechanisms in aquatic systems and are used to determine the fate factor used to calculate the midpoint characterization factor in work by Corella-Puertas et al. for expanded polystyrene and tire and road wear particles. Including microplastic impacts in LCA damage assessments was found to be significant for expanded polystyrene, though differing polymer degradation rates found in the literature result in high uncertainty. In the case of tire particles, sedimentation rates were the dominant mechanism vs. degradation, though the degradation data used were from soil studies, not marine aquatic environments. Schwartz et al. [30] extended this approach to LDPE, PP, and PET and echoed the need for more data regarding fragmentation and mineralization. Though Corella-Puertas et al. provide a method for determining midpoint and endpoint characterization factors, their results highlight the need for experimental measurements and modeling to improve LCA accuracy and precision.

One of the hallmarks of compostable materials is that the generation of microplastics from them is not persistent, lessening their impact on the environment [12,31]. This persistence can be understood by their level of biodegradation, which has standards for measurement, e.g., ASTM D6400, ISO 17088, and EN 13432 [11,32,33]. When materials are composted in appropriate environments, the issue of persistence is eliminated. Work on aromatic–aliphatic compostable polyesters during industrial composting has shown that plastic fragmentation is expected, but no accumulation for all sizes measured was detected [34,35]. Additional work for other compostable polyesters found similar findings that showed when in the presence of water/humidity; compostable plastics would not leave persistent pollution unlike non-biodegradable plastics [36].

**Figure 7:**
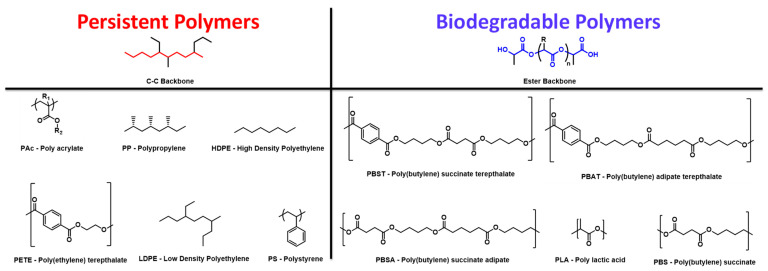
Chemical structures of common persistent polymers and biodegradable polymers.

It is important to understand how end-of-life scenarios differ as well for non-compostable C-C bond polymers (Figure 7). Landfilling is a well-known contributor to both bulk plastic waste generation and microplastic generation. Recent studies, however, speak to the realities of mechanical recycling and incineration as point sources of microplastic generation, which has largely been overlooked when discussing plastic waste. While mechanical recycling is likely to increase as we move to a circular plastics economy, greater microplastic emissions can be expected based on their origin coming from recycled material [37]. When it comes to incineration, it is widely accepted that incineration can permanently eliminate bulk plastic waste. This understanding naturally carries over to microplastics as well; however, unburned material still exists in the bottom ash, which is a solid residue from incinerators. A study investigating this bottom ash discovered that microplastics were present and served as a neglected microplastic source with an abundance of 1.9–565 n/kg, which indicated that each metric ton of waste produces 360 to 102,000 microplastic particles after incineration [38]. While one can expect each end-of-life to largely affect the types of plastic waste produced (bulk and microplastic), composting exists as a solution, which can eliminate microplastic persistence and bulk plastic accumulation when industrially composted.

### 4.3. Microplastics: Composting as a Proposed Solution to Microplastic Persistence

When plastics persist in the environment, they can break down into micron and sub-micron materials, which are found in many environmental settings including aquatic and terrestrial environments [39,40]. The prevalent shapes of microplastics, in water and sediments, were found to be fragments and fibers. Moreover, the particle size of microplastics is mainly less than 2 mm, with PP and PE consisting of the main polymer types in the freshwater environment of China. The authors have confirmed that leakage into land and ocean environments is a major contributor and that microplastics can be relocated by atmospheric long-range transport. As such, the site of landfilling/contamination is not where microplastics are restricted and are able to pollute distant places. LDPE is the most abundant source of microplastic production worldwide, and its persistence and presence has been measured in terrestrial settings with uptake into worms [41]. One of the challenges with microplastics deals with correlating their abundance and size distribution with associated health risks. Modeling offers a chance to understand source identification, transport, and risk assessment related to microplastics [42]. With better prediction and quantification methods for microplastics, the number of research articles has greatly expanded, especially as it relates to humans.

### 4.4. Microplastics: Identification and Toxicological Measurements

**Figure 8:**
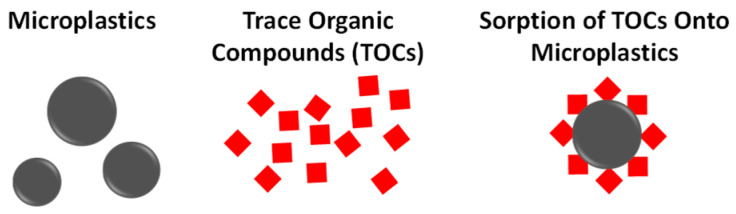
Relationship between microplastics and trace organic compounds, which have an associated toxicity. Sorption onto microplastics affords a jointly toxic carrier, which leads to higher levels of toxic exposure.

Based on the accumulation of microplastics that arise from non-compostable plastics, a leading concern is their presence in food, animals, and even humans. While a human health risk assessment for plastic particle pollution is currently not possible due to a lack of data on both toxicological hazard and human exposure, recent reports are accelerating the research in this area by establishing methods to detect this pollution [6,7]. Researchers note that respiratory symptoms and disease following exposure to occupational levels of microplastics within industry settings have been reported, and being able to track and quantify these contaminants is necessary. These studies reveal the persistence of microplastics, which is measurable in human blood and in the lungs, and questions relating to the impact on health and immune regulation in humans is now an apparent research area to explore for microplastic toxicity (Figure 8). The most prevalent microplastics found in the blood included polyethylene, polyethylene terephthalate, and polystyrene [6]; the most prevalent microplastics found in human lungs included both polypropylene and polyethylene terephthalate. While the risk is not fully understood for humans, work has identified that differences in size and types of plastic pollution have impacts in terms of toxicity.

Studies with snails showed that smaller-sized particles of polyethylene triggered oxidative stress compared to larger-sized ones [43]. Polystyrene microplastics (PS-MPs) were shown to also display a difference in toxicity impact based on size. While PS-MPs, with sizes ranging from 1–10 μm and 50–100 μm, both resulted in necroptosis and inflammation to mice bladder epithelium, smaller PS-MPs proved to be more severe in necroptosis while larger PS-MPs led to a higher degree of inflammation [44]. In another study, which looked at PVC microplastic toxicity in human and fish lymphocytes, cytotoxicity was induced in their presence [45]. Based on their findings, human lymphocytes were more sensitive to PVC microplastics than fish lymphocytes. This toxicity difference between bulk plastic and the microplastics they generate is gaining more traction as evidenced by the large body of work coming out in just the past 2 years. A recent finding also revealed that microplastics themselves are not only toxic due to their size and makeup but also the interaction they have with other toxins (Figure 8). It has been reported that toxic organic compounds (TOCs) and polystyrene microplastics had a minor toxic effect on Caco-2 cells when tested separately, whereas TOC-sorbed microplastics at a similar concentration have an order of magnitude higher toxicity than pristine microplastics [46]. This joint toxic discovery demonstrates the real-world scenario in which the co-existence of these contaminants can lead to elevated risks for humans and their environment. Table 2 captures some of the health risks associated with the most common types of plastics, and a more in-depth study of microplastic toxicity for mammal cell lines has recently been published [47].

A recent comprehensive review of the effect that microplastics have on human cell lines and model organisms was published [48]. They investigated the effects of polystyrene, polypropylene, polyethylene, polyvinyl chloride, polymethyl methacrylate, and polyethylene terephthalate, which all ranged in sizes from 40 nm up to 150 µm. A range of biological effects was measured ranging from cell stress to apoptotic increase. The author’s goal was to contribute to the establishment of micro- and nanoplastic regulation protecting global food safety and security. A recent article summarized the health effects measured directly for humans [49]. From the retrospective studies and case reports covered, they were able to identify eight human health systems, which were affected by microplastics. Some to note include genetoxicity, which was observed in workers exposed to styrene, as well as osteolysis around bone prostheses linked to PE particles. While there are currently limited human studies in this area, positive correlations between MPs are being reported for human health. We foresee the emphasis on microplastic generation, persistence, bioaccumulation, and toxicity to increase in the following years, while their incorporation into LCAs becomes more pervasive. For now, summarizing and including related works for plastics, which accumulate in the environment, serves as a transient action to highlight the benefits of composting until LCAs can document the impacts that are being established for microplastics.

**Table 2:** Associated health risks of microplastics with fish species.

Polymer Type	Size Distribution	Toxicity Effect(s)	Study
Polyethylene	Micron Fragments	Liver and gill histopathology Changes in blood biochemistry Changes in the expression of reproductive axis genes	[50]
	~70 µm	Intestinal damage of adult fish gut	[51]
	40–150 µm	Increased oxidative burst of in leukocytes of Sparus aurata Upregulation of the redox regulator Nrf2 in leukocytes of Sparus aurata	[52]
	250 and 500 μg/L	Changes in plasma levels of various metabolic enzymes and immune markers Combination of MP and Cd increased Cd toxicity	[53]
Polypropylene	~70 µm	Intestinal damage of adult fish gut	[54]
Polystyrene	24 and 27 nm	Defects in metabolism, changes in brain appearance and weight	[55]
	5 µm + cadmium	Increased Cd accumulation in livers, guts, and gills, Enhanced Cd toxicity, Combined exposure caused oxidative damage and inflammation in tissues	[56]
	52 µm (amino modified)	Changes in feeding time Changes in brain morphology (gyri sizes)	[57]

## 5. Conclusions

This LCA study has shown that carbon sequestration is an integral part of accounting for carbon dioxide emissions of bio-based systems and should be accurately depicted in all system boundaries. PLA represents a bio-based material whose GWP was identified to be 12× less of an emitter compared to LDPE, and this is only captured in a cradle-to-gate analysis when carbon sequestration is counted. It is imperative that all LCA methods adopt these accounting methods moving forward so that more concrete decisions can be made for biobased polyesters to replace carbon-rich fossil-based plastics. In addition to the comparison of biobased and fossil-based plastics, we anticipate the same discussions to be had around compostable and non-compostable materials. As highlighted in the recent literature surrounding microplastics and polymer persistence, composting should be adopted as a solution to avoid the generation of microplastics. The detection of microplastics is in its nascent stages, yet work has already been able to document direct toxicity based on particle size, and joint toxic effects that exist between toxic organic compounds, which are concentrated onto microplastics. LCAs still lack proper accounting for these rising issues, yet the continued global understanding of detecting microplastics and assessing their toxicological impact will inevitably solidify their accounting in LCA methodologies moving forward.

## Figures and Tables

**Table 1:** Material carbon footprint.

LDPE Gloves	LDPE Gloves	NT Gloves
Unit of Measure	Value	Value
Length (m)	1550.00	1550.00
Width (mm)	320.00	320.00
Thickness (μm)	19.85	18.00
Density (g/cc)	0.93	1.25
Weight of one roll (kg)	18.31	22.32
Gloves/roll	5000.00	5000.00
Weight of film per glove (g)	3.66	4.46
Total film weight for gloves—functional unit (Tons)	3.66	4.46
Carbon Content (%)	86	60
Bio-Content (%)	0	21
Total CO_2_ evolution (Tons)	11.51	7.72

## Data Availability

The original contributions presented in this study are included in the article/Supplementary Materials. Further inquiries can be directed to the corresponding authors.

## Supplementary Materials

The following supporting information can be downloaded at https://www.mdpi.com/article/10.3390/polym16213073/s1: Figure S1: Sensitivity Analysis for GWP, ME, TE and AP; Figure S2: Sensitivity Analysis for PM, NRE, RE, and ODP; Figure S3: Normalized Impacts for GWP, ME, TE and AP; Figure S4: Normalized Impacts for PM, NRE, RE, and ODP. Table S1: Life Cycle Inventory for NT Gloves—Inputs; Table S2: Life Cycle Inventory for NT Gloves—Measured Impact Categories; Table S3: Life Cycle Inventory for LDPE Gloves—Inputs; Table S4: Life Cycle Inventory for LDPE Gloves—Measured Impact Categories; Table S5: Life Cycle Inventory Table for PLA and Copolyester; Table S6: Life Cycle Inventory Table for LDPE.

## Abbreviations

AP	acidification potential
GREET	greenhouse gases, regulated emissions, and energy use in technologies
GWP	global warming potential
HDPE	high-density polyethylene
JRC	joint research center
LCA	life cycle assessment
LCIA	life cycle impact assessment
LDPE	low-density polyethylene
ME	marine eutrophication
MP	Microplastic
NRE	non-renewable enegry
ODP	ozone depletion potential
PEF	product environmental footprint
PET	polyethylene terephthalate
PLA	polylactic acid
PM	particulate matter
PP	polypropylene
PS-MPs	polystyrene microplastics
RE	renewable energy
TE	terrestrial eutrophication
TOC	trace organic compounds
TRACI	tool for reduction and assessment of chemicals and other environmental impacts
W2W	wheel to wheel
