# Supplementary material for: Biobased Compostable Plastics End-of-Life: Environmental Assessment Including Carbon Footprint and Microplastic Impacts
Source: Polymers (Basel). 2024 Oct 31;16(21):3073. doi: 10.3390/polym16213073 (PMC11548190; doi:10.3390/polym16213073)
Supplement: Supplementary file 1 [file polymers-16-03073-s001.zip › polymers-3281388-supplementary.pdf]

Supporting Information

Biobased Compostable Plastics End-of-Life: Environmental Assessment Including Carbon Footprint and Microplastic Impacts

Anthony Keyes[a*], Christopher M. Saffron[b], Shilpa Manjure[a], Ramani Narayan[b]

[a] *Northern Technologies International Corporation, Natur-Tec, 4201 Woodland Road, Circle Pines, MN, 55014*

[b] *Michigan State University, 204 Farrall Hall, East Lansing, MI 48824*

Contents

Life Cycle Inventory Data Sheets 3
Table S1. Life Cycle Inventory for NT Gloves - Inputs 3
Table S2. Life Cycle Inventory for NT Gloves – Measured Impact Categories 4
Table S3. Life Cycle Inventory for LDPE Gloves - Inputs 5
Table S4. Life Cycle Inventory for LDPE Gloves – Measured Impact Categories 6
Data Quality Indicators 7
Table S5. Life Cycle Inventory Table for PLA and Copolyester 7
Table S6. Life Cycle Inventory Table for LDPE 8
Sensitivity Analysis 9
Comparative Charts 11
Conversion Factors 13
Converting Emissions to Impact Category 13
Normalization Calculation for US Citizen Per Capita 13

Life Cycle Inventory Data Sheets

Life cycle inventory data sheets document the amount of resin, water, electricity, travel and packaging used to generate 1,000,000 gloves. The steam numbers for Tables S1-S4 correlated to the system boundaries of the system (Figure 4-5 in paper).

Table S1. Life Cycle Inventory for NT Gloves - Inputs

	Inputs				
	Resin	*Water*	*Electricity*	*Distance*	*Material Packaging*
**Stream #**	*kgs*	*liters*	*kWh*	*km*	*kgs*
**PLA Impact Categories**	***1.00***	-	-	-	-
**Copolyester Impact Categories**	***1.00***	-	-	-	-
**Electricity (Tamil Nadu)**	-	-	***1.00E+00***	-	-
**Short Haul Truck**	***1000.00***	-	-	***1.00E+00***	-
**Ship**	***1000.00***	-	-	***1.00E+00***	-
**Rail Freight Diesel**	***1000.00***	-	-	***1.00E+00***	-
**1**	207.39				
**2**	892.00				
**3**	165.02				
**4**	3122.00				
**5**	57.98				
**6**	15.61				
**7**	446.00				
**8**		223	2.28E+02		
**9**					
**10**	446.00				
**11**		1.50E-01			
**12**		1.50E-01			
**13**	446.00				
**14**			1.42E+00		
**15**					
**16**	446.00				
**17**					6.07E+00
**18**	4460.00				
**19**		1.06E+03	1.28E+03		
**20**					
**21**	4460.00				
**22**			2.14E+02		
**23**					
**24**	4460.00				
**25**			3.64E+02		
**26**					
**27**	4460.00				
**28**					3.17E+02
**29**	207.39			4.98E+03	
**30**	165.02			1.50E+04	
**31**	892.00			4.98E+03	
**32**	3122.00			4.98E+03	
**33**	446.00			0.00E+00	
**34**	4460.00				
**Total**	4460.00	1.28E+03	2.09E+03	2.99E+04	3.23E+02

Stream # correlates to the system boundaries (located in the appendix). Stream categories highlighted in **blue** show the six different inputs which are being measured to determine the output for measured impact categories. The functional unit for this table is 1,000,000 gloves which resulted in a total mass output of 4460 kgs.

Table S2. Life Cycle Inventory for NT Gloves – Measured Impact Categories

	**Measured Impact Categories**							
	GWP	***ME***	***TE***	***AP***	***PM***	***NREU***	***REU***	***ODP***
**Stream #**	*kg CO_2 eq.*	*kg of N eq.*	*mol N eq.*	*mol H + eq.*	*kg PM 2.5 eq.*	*GJ*	*GJ*	kg CFC-11 eq.
**PLA Impact Categories**	***5.01E-01***	***1.33E-02***	***3.38E-02***	***1.82E-02***	***1.74E-03***	***2.88E-02***	***6.04E-02***	***0.00E+00***
**Copolyester Impact Categories**	***3.70E+00***	***1.40E-03***	***1.51E-02***	***5.17E-03***	***2.79E-04***	***1.18E-03***	***7.57E-05***	***6.66E-11***
**Electricity (Tamil Nadu)**	***3.98E-01***	***8.68E-05***	***5.70E-04***	***4.90E-05***	***9.33E-06***	***1.80E-03***	***1.80E-03***	***0.00E+00***
**Short Haul Truck**	***9.45E-02***	***4.27E-06***	***2.81E-05***	***1.22E-06***	***5.18E-05***	***1.39E-03***	***0.00E+00***	***0.00E+00***
**Ship**	***1.09E-04***	***0.00E+00***	***1.42E-06***	***7.61E-08***	***2.53E-07***			***0.00E+00***
**Rail Freight Diesel**	***1.77E-02***	***0.00E+00***	***3.66E-05***	***1.33E-06***	***1.45E-06***			***0.00E+00***
**1**	1.04E+02	2.76E+00	7.01E+00	3.77E+00	3.61E-01	5.97E+00	1.25E+01	0.00E+00
**2**	4.47E+02	1.19E+01	3.01E+01	1.62E+01	1.55E+00	2.57E+01	5.39E+01	0.00E+00
**3**	6.11E+02	2.31E-01	2.49E+00	8.53E-01	4.60E-02	1.95E-01	1.25E-02	1.10E-08
**4**	1.16E+04	4.37E+00	4.71E+01	1.61E+01	8.71E-01	3.68E+00	2.36E-01	2.08E-07
**5**								
**6**								
**7**								
**8**								
**9**	9.10E+01	1.98E-02	1.30E-01	1.12E-02	2.13E-03	4.11E-01	4.11E-01	0.00E+00
**10**								
**11**								
**12**								
**13**								
**14**								
**15**	5.67E-01	1.24E-04	8.12E-04	6.97E-05	1.33E-05	2.56E-03	2.56E-03	0.00E+00
**16**								
**17**								
**18**								
**19**								
**20**	5.12E+02	1.11E-01	7.33E-01	6.29E-02	1.20E-02	2.31E+00	2.31E+00	0.00E+00
**21**								
**22**								
**23**	8.53E+01	1.86E-02	1.22E-01	1.05E-02	2.00E-03	3.85E-01	3.85E-01	0.00E+00
**24**								
**25**								
**26**	1.45E+02	3.16E-02	2.08E-01	1.78E-02	3.40E-03	6.55E-01	6.55E-01	0.00E+00
**27**								
**28**								
**29**	2.47E-01	1.12E-05	7.34E-05	3.20E-06	1.35E-04	3.64E-03	0.00E+00	0.00E+00
**30**	6.84E+00	2.95E-04	5.46E-03	2.72E-04	4.19E-03	9.61E-02	0.00E+00	0.00E+00
**31**	1.06E+00	4.80E-05	3.16E-04	1.37E-05	5.82E-04	1.57E-02	0.00E+00	0.00E+00
**32**	1.29E+02	5.57E-03	1.03E-01	5.14E-03	7.92E-02	1.82E+00	0.00E+00	0.00E+00
**33**								
**34**								
**Total**	1.37E+04	1.94E+01	8.81E+01	3.71E+01	2.93E+00	4.12E+01	7.04E+01	2.19E-07

Stream # correlates to the system boundaries (located in the appendix). Stream categories highlighted in **blue** show the six different streams whose measured impact categories are populated per unit measurement. These steam categories are used to populate the stream table by multiplying the inputs by their according stream category. The functional unit for this table is 1,000,000 gloves which resulted in a total mass output of 4460 kgs.

Table S3. Life Cycle Inventory for LDPE Gloves - Inputs

	Inputs				
	Resin	***Water***	***Electricity***	***Distance***	***Material Packaging***
**Stream #**	*kgs*	*liters*	*kWh*	*km*	*kgs*
**LDPE Impact Categories**	***1.00***	-	-	-	-
**Electricity (Tamil Nadu)**	-	-	***1.00E+00***	-	-
**Long Haul Truck**	***1000.00***	-	-	***1.00E+00***	-
**1**	135.55				
**2**	3480.39				
**3**	47.63				
**4**	183.18				
**5**		91.58929	9.38E+01		
**6**					
**7**	183.18				
**8**		1.50E-01			
**9**		1.50E-01			
**10**	183.18				
**11**			5.85E-01		
**12**					
**13**	183.18				
**14**					2.49E+00
**15**	3663.57				
**16**		8.72E+02	1.06E+03		
**17**					
**18**	3663.57				
**19**			1.76E+02		
**20**					
**21**	3663.57				
**22**			2.99E+02		
**23**					
**24**	3663.57				
**25**					2.60E+02
**26**	135.55			6.71E+02	
**27**	3663.57			6.71E+02	
**28**	183.18			0.00E+00	
**29**	3663.57				
**Total**	3663.57	9.64E+02	1.62E+03	1.34E+03	2.63E+02

Stream # correlates to the system boundaries (located in the appendix). Stream categories highlighted in **blue** show the six different inputs which are being measured to determine the output for measured impact categories. The functional unit for this table is 1,000,000 gloves which resulted in a total mass output of 3664 kgs.

Table S4. Life Cycle Inventory for LDPE Gloves – Measured Impact Categories

	Measured Impact Categories							
	GWP	*ME*	*TE*	*AP*	*PM*	*NREU*	*REU*	*ODP*
**Stream #**	*kg CO_2 eq.*	*kg of N eq.*	*mol N eq.*	*mol H + eq.*	*kg PM 2.5 eq.*	*GJ*	*GJ*	kg CFC-11 eq.
**LDPE Impact Categories**	***1.93E+00***	***3.00E-04***	***6.10E-04***	***6.50E-03***	***9.97E-04***	***7.98E-02***	***5.00E-04***	***1.30E-06***
**Electricity (Tamil Nadu)**	***3.98E-01***	***8.68E-05***	***5.70E-04***	***4.90E-05***	***9.33E-06***	***1.80E-03***	***1.80E-03***	***0.00E+00***
**Long Haul Truck**	***6.15E-02***	***5.29E-06***	***3.48E-05***	***1.30E-06***	***8.75E-07***	***1.39E-03***	***0.00E+00***	***0.00E+00***
**1**	2.61E+02	4.07E-02	8.26E-02	8.81E-01	1.35E-01	1.08E+01	6.78E-02	1.76E-04
**2**	6.71E+03	1.04E+00	2.12E+00	2.26E+01	3.47E+00	2.78E+02	1.74E+00	4.52E-03
**3**								
**4**								
**5**								
**6**	3.74E+01	8.14E-03	5.35E-02	4.59E-03	8.75E-04	1.69E-01	1.69E-01	0.00E+00
**7**								
**8**								
**9**								
**10**								
**11**								
**12**	2.33E-01	5.07E-05	3.34E-04	2.86E-05	5.46E-06	1.05E-03	1.05E-03	0.00E+00
**13**								
**14**								
**15**								
**16**								
**17**	4.20E+02	9.15E-02	6.02E-01	5.17E-02	9.84E-03	1.90E+00	1.90E+00	0.00E+00
**18**								
**19**								
**20**	7.01E+01	1.53E-02	1.00E-01	8.61E-03	1.64E-03	3.17E-01	3.17E-01	0.00E+00
**21**								
**22**								
**23**	1.19E+02	2.59E-02	1.71E-01	1.46E-02	2.79E-03	5.38E-01	5.38E-01	0.00E+00
**24**								
**25**								
**26**	5.59E+00	4.81E-04	3.16E-03	1.18E-04	7.96E-05	1.27E-01	0.00E+00	0.00E+00
**27**	1.51E+02	0.00E+00	1.92E-02	0.00E+00	6.17E-08	0.00E+00	0.00E+00	0.00E+00
**28**								
**29**								
**Total**	7.77E+03	1.23E+00	3.15E+00	2.36E+01	3.62E+00	2.92E+02	4.73E+00	4.70E-03

Stream # correlates to the system boundaries (located in the appendix). Stream categories highlighted in **blue** show the six different streams whose measured impact categories are populated per unit measurement. These steam categories are used to populate the stream table by multiplying the inputs by their according stream category. The functional unit for this table is 1,000,000 gloves which resulted in a total mass output of 3364 kgs.

Data Quality Indicators

Data quality indicators were benchmarked utilizing the Weidima method.

Table S5. Life Cycle Inventory Table for PLA and Copolyester

Variable Inputs	Unit of Measure	Value	Data Source	DQI A	B	C	D	E
**Resins**								
PLA Resin	% Mass	25.00	doi.org/10.1007/s10924-019-01525-9	1	1	1	1	1
Copolyester Resin	% Mass	75.00	Supplier (Confidential)	2	1	2	3	5
**Reactive Extrusion Process**								
Water for Cooling Extruder	L / kg material	0.50	Directly Measured	1	1	1	1	1
Water for Water Bath	L / run	0.15	Directly Measured	1	1	1	1	1
Electricity for Extruder	kWh / kg material	0.51	Directly Measured	1	1	1	1	1
Electricity for Pelletizer	kWh / kg material	0.00	Directly Measured	1	1	1	1	1
Vaccum Electricity Demand	kWh / kg material	0.04	Directly Measured	1	1	1	1	1
Oven Electricity Demand	kWh / kg material	0.03	Directly Measured	1	1	1	1	1
Packaging of Granulate	kg packaging / kg material	0.01	Directly Measured	1	1	1	1	1
**Blown Film Extrusion Process**								
Water for Cooling Extruder	L / kg material	0.24	Directly Measured	1	1	1	1	1
Electricity for Extruder	kWh / kg material	0.29	Directly Measured	1	1	1	1	1
Electricity for Air Flow	kWh / kg material	0.05	Directly Measured	1	1	1	1	1
Rolling Electricity Demand	kWh / kg material	0.08	Directly Measured	1	1	1	1	1
Oven Electricity Demand	kWh / kg material	0.05	Directly Measured	1	1	1	1	1
Packaging of Granulate	kg packaging / kg material	0.07	Directly Measured	1	1	1	1	1
**Transportation**								
PLA Transport								
1) Supplier to Port	km	12.60	GREET 2021 (Short Haul Truck)	2	1	2	3	3
2) Port to Port	km	4950.40	doi.org/10.1007/978-3-319-30127-3_65	2	2	1	2	1
3) Port to India	km	19,8	GREET 2021 (Short Haul Truck)	2	1	2	3	3
Copolyester Transport								
1) Supplier to Port	km	418.00	GREET 2021 (Rail Freight Diesel)	2	1	2	3	3
2) Port to Port	km	14553.00	doi.org/10.1007/978-3-319-30127-3_65	2	2	1	2	1
3) Port to India	km	19.80	GREET 2021 (Short Haul Truck)	2	1	2	3	3

Table S6. Life Cycle Inventory Table for LDPE

Variable Inputs	Unit of Measure	Value	Data Source	DQI A	B	C	D	E
**Resins**								
LDPE Resin	% Mass	100.00	cradle-to-gate-life-cycle-analysis-of-LDPE	2	1	1	1	1
**Reactive Extrusion Process**								
Water for Cooling Extruder	L / kg material	0.50	Directly Measured	1	1	1	1	1
Water for Water Bath	L / run	0.15	Directly Measured	1	1	1	1	1
Electricity for Extruder	kWh / kg material	0.51	Directly Measured	1	1	1	1	1
Electricity for Pelletizer	kWh / kg material	0.00	Directly Measured	1	1	1	1	1
Vaccum Electricity Demand	kWh / kg material	0.04	Directly Measured	1	1	1	1	1
Oven Electricity Demand	kWh / kg material	0.03	Directly Measured	1	1	1	1	1
Packaging of Granulate	kg packaging / kg material	0.01	Directly Measured	1	1	1	1	1
**Blown Film Extrusion Process**								
Water for Cooling Extruder	L / kg material	0.24	Directly Measured	1	1	1	1	1
Electricity for Extruder	kWh / kg material	0.29	Directly Measured	1	1	1	1	1
Electricity for Air Flow	kWh / kg material	0.05	Directly Measured	1	1	1	1	1
Rolling Electricity Demand	kWh / kg material	0.08	Directly Measured	1	1	1	1	1
Oven Electricity Demand	kWh / kg material	0.05	Directly Measured	1	1	1	1	1
Packaging of Granulate	kg packaging / kg material	0.07	Directly Measured	1	1	1	1	1
**Transportation**								
LDPE Transport Supplier to NT India	km	671.00	GREET 2021 (Long Haul Truck)	2	1	2	3	3

Sensitivity Analysis

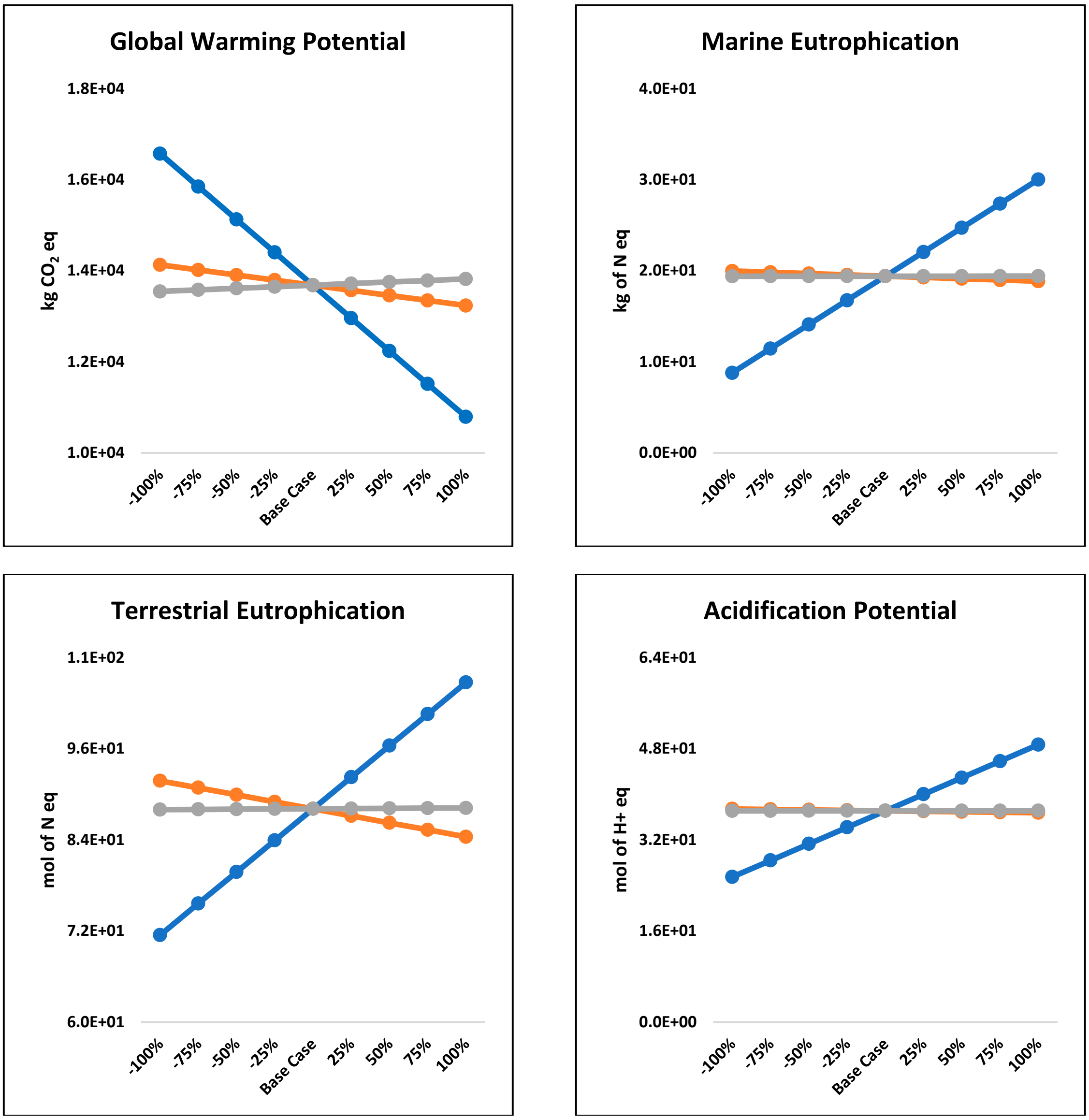

Figure S1. Sensitivity analysis for the measured impact categories (GWP, ME, TE and AP). The **blue** line measures the change in the ratio of PLA:Copolyester used in the formulation. The **orange** line refers to the energy input ratio for REU:NREU used in the system. Lastly, the **grey** line refers to the distance required for the total transportation of materials.

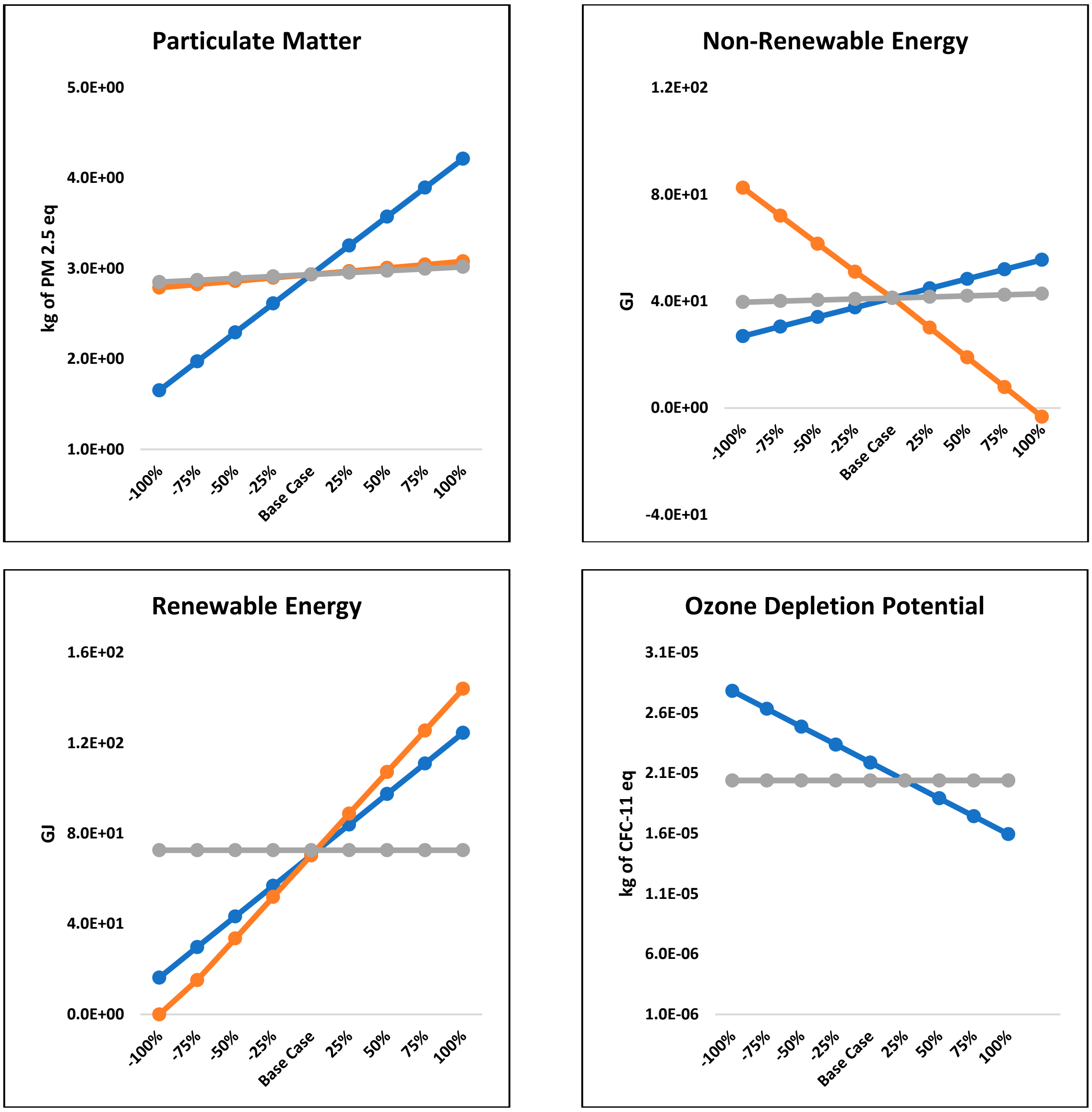

Figure S2. Sensitivity analysis for the measured impact categories (PM, NREU, REU and ODP). The blue line measures the change in the ratio of PLA:Copolyester used in the formulation. The orange line refers to the energy input ratio for REU:NREU used in the system. Lastly, the grey line refers to the distance required for the total transportation of materials.

Comparative Charts

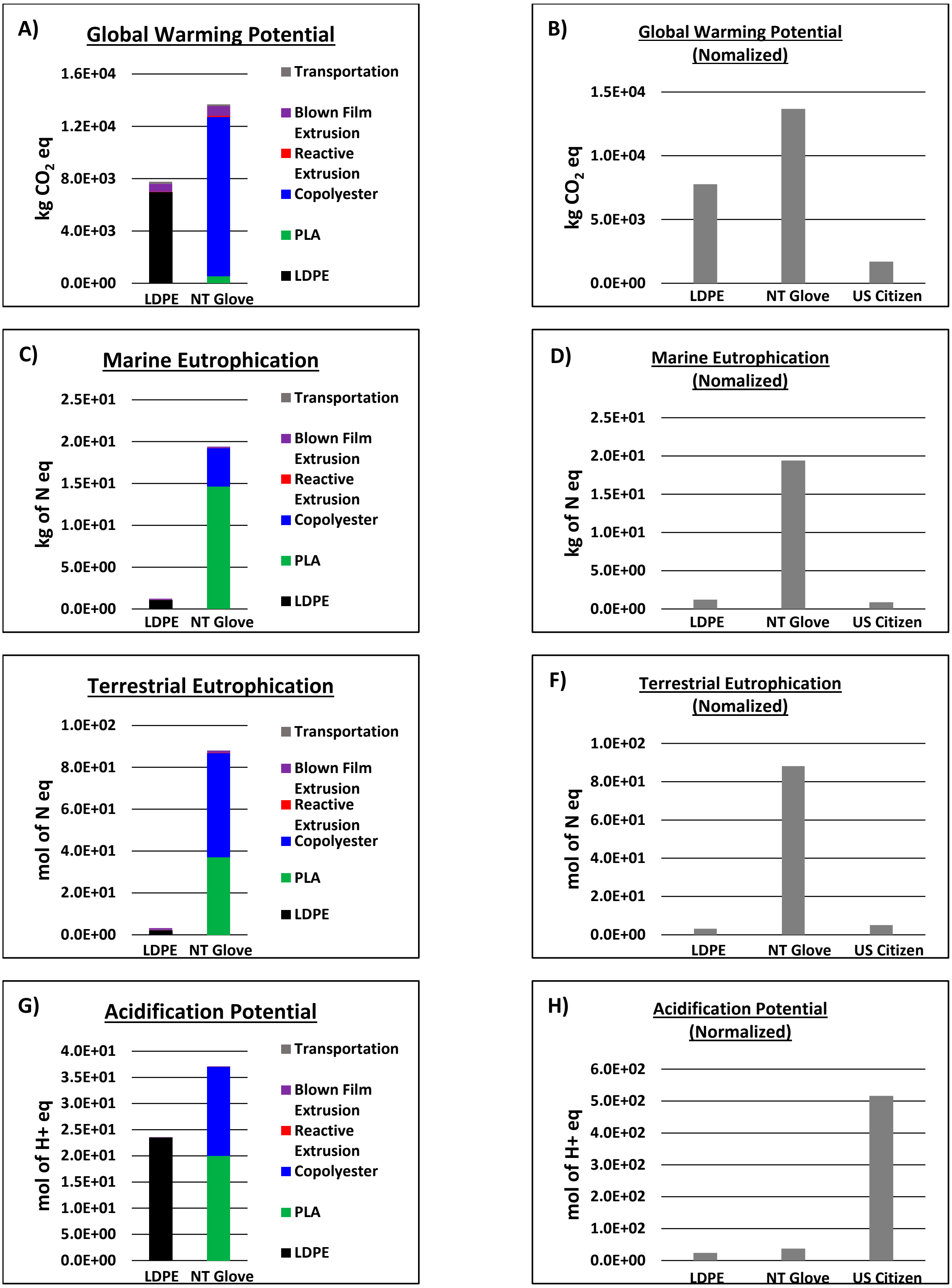

Figure S3: Stacked bar charts showing the environmental impacts (GWP, ME, TE, and AP) for LDPE and NT gloves. Normalized bar charts are displayed on the right on the stacked bar chart categories.

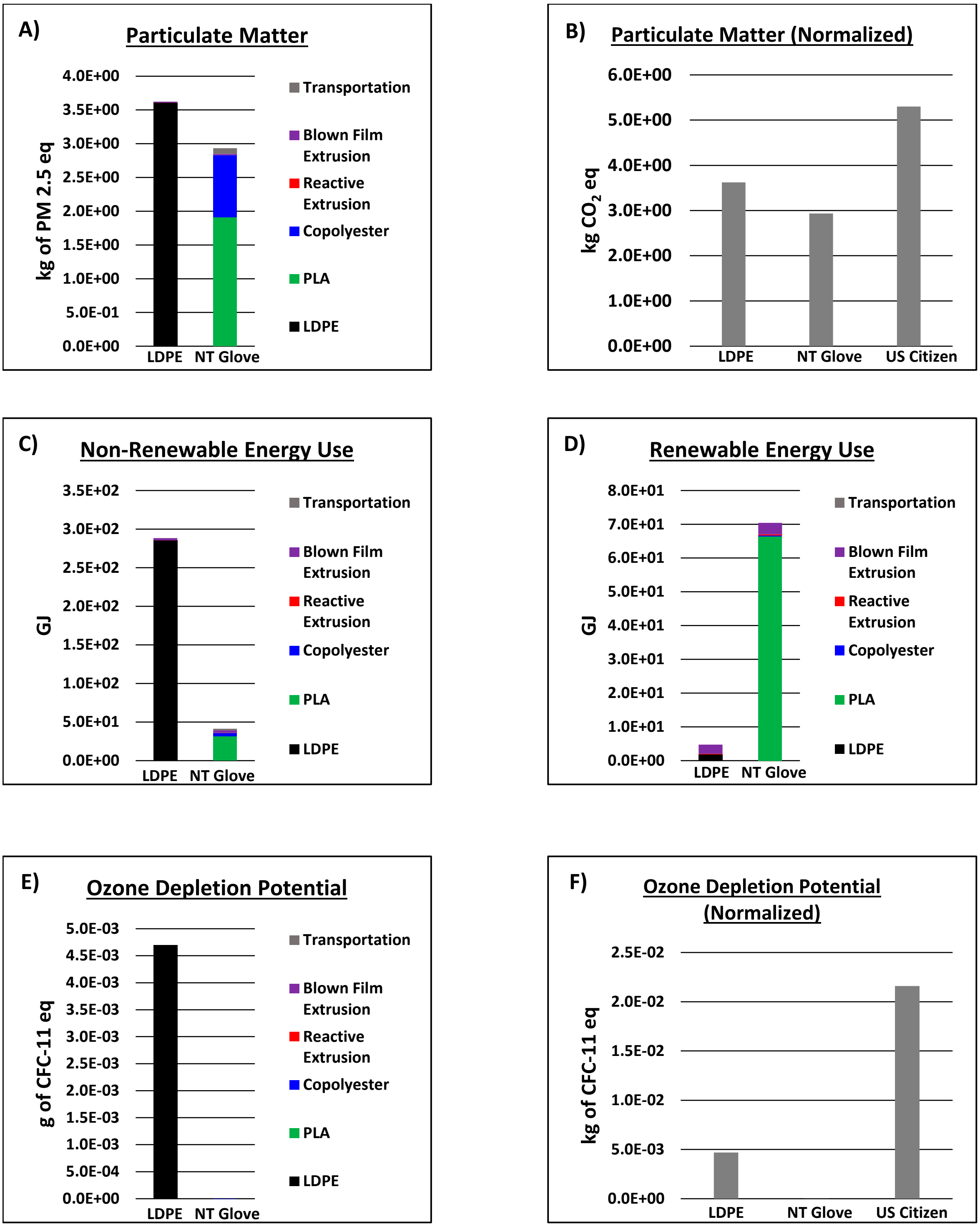

Figure S4: Stacked bar charts showing the environmental impacts (PM, NREU, REU and ODP) for LDPE and NT gloves. Normalized bar charts are shown for PM and ODP on the right of the stack bar charts.

Conversion Factors

Converting Emissions to Impact Category

TRACI data were used to convert measured emissions into equivalent values for impact categories (Bare, 2008). The following calculations were used.

- GWP Characterization Factors
  - 1 kg of CH_4 = 25 kg of CO_2 equivalents
  - 1 kg of N_2O = 298 kg of CO_2 equivalents
- ME Characterization Factors
  - 1 kg of NO_x = 0.0686 kg of N equivalents
- TE Characterization Factors
  - 1 kg of NO_x = 0.451 mol of N equivalents
- AP Characterization Factors
  - 1 kg of NO_x = 0.016195925 mol of H^+ equivalents
  - 1 kg of SO_x = 0.015609 mol of H^+ equivalents

Example Calculation: 10 kg of CH_4 = (10*25) kg CO_2 equivalents = 250 kg CO_2 equivalents

Normalization Calculation for US Citizen Per Capita

- Functional Unit = 1,000,000 gloves
- Annual glove production = 14,400,000 gloves / year
- Conversion factor = 0.069444 years

Example Calculation: Comparing 10 kg CO_2 equivalents of material to (1000 kg CO_2 equivalents by US citizen / year)

- (1000 kg CO_2 equivalents by US citizen / year)*(0.069444 years)
  - = 69.44 kg CO_2 equivalents by US citizen
- Normalized Factor = 10 kg CO_2 equivalents of material / 69.44 kg CO_2 equivalents by US citizen
  - = 0.144 normalized factor of material to a US citizen
